# Human models for human diseases: how pluripotent stem cell derived brain organoids can help us understand and treat diffuse hemispheric glioma

**DOI:** 10.3389/fcell.2026.1962790

**Published:** 2026-10-28

**Authors:** Patrick L. Barry, Dushana M. Alagiyawanna, Rhiannon B. Werder, Maree C. Faux, David D. Eisenstat

**Affiliations:** 1 Stem Cell Medicine, Murdoch Children’s Research Institute, Parkville, VIC, Australia; 2 Department of Paediatrics, Faculty of Medicine, Dentistry and Health Sciences, University of Melbourne, Parkville, VIC, Australia; 3 Novo Nordisk Foundation Centre for Stem Cell Medicine, reNEW Melbourne, Parkville, VIC, Australia; 4 Department of Surgery, Faculty of Medicine, Dentistry and Health Sciences, University of Melbourne, Melbourne, VIC, Australia; 5 Children’s Cancer Centre, The Royal Children’s Hospital Melbourne, Parkville, VIC, Australia

**Keywords:** 3D brain organoids, diffuse hemispheric glioma, epigenetics, GABAergic interneuron, histone H3.3 G34 mutation, iPSC (induced pluripotent stem cell)

## Abstract

The precise orchestration of neurodevelopment and epigenetic regulation is essential for normal brain development with devastating consequences if disrupted, leading to neurological diseases and cancer. For diseases such as paediatric high grade gliomas (pHGG), the epigenetic driver mutation in Histone 3.3 contorts the fate of developing cells in the brain, resulting in poor outcomes for most children and adolescents with this disease. The complexity of diseases such as pHGG is reflected in the intricacy of the development of human cortical structures and their subsequent neural functions; it is our cortex that significantly delineates us from all other mammalian species. This complexity cannot be modelled adequately in systems other than human. Indeed, there are thousands more neurons with a thousand-fold increase in surface area of the human brain in comparison to the mouse cortex. Approaches to study the human developing brain are therefore required to understand the complexities of human cortical structures. Here, we describe the neurodevelopmental trajectories and epigenetic regulation of human cortex development. We then report how investigators study the human cortex, how it can be recapitulated in 3D brain organoids, and why this is important for modelling disease. We focus on the lethal childhood brain cancer H3G34-mutated Diffuse Hemispheric Glioma (DHG), a sub-type of pHGG arising in the cortex of adolescents, as an exemplar for modelling a difficult-to-treat disease in 3D brain organoids. Finally, we provide a perspective on the current shift from research using animals to New Approach Methodologies (NAMs) and why 3D brain organoids offer opportunities for the future of pre-clinical validation.

## Introduction

Central nervous system (CNS) tumours are the leading cause of cancer related deaths in children (Ostrom et al., 2020), of which paediatric high-grade gliomas (pHGG) include some of the most aggressive subtypes and are classified by the World Health Organisation as grade 4 tumours (Bavle and Chintagumpala, 2018). More than 30% of pHGG are classified as diffuse hemispheric glioma (DHG), H3.3 G34 mutant, and arise in the cerebral hemispheres of adolescents and young adults (Buccoliero et al., 2022). DHG is molecularly defined by a heterozygous mutation in the *H3-3A* (formerly *H3F3A)* gene which encodes the histone variant H3.3, resulting in the substitution of a glycine for either an arginine, or less frequently valine, at residue 34 (Schwartzentruber et al., 2012). DHG onset ranges from 13–22 years old and the median survival rate ranges from 15–22 months post-treatment (Crowell et al., 2022). The driver mutation in DHG occurs in *H3-3A*/*H3F3A* and results in expression of the H3.3 G34R/V, often accompanied by co-mutations such as loss of *TP53*, *ATRX* (over 90% of cases) and amplification of *PDGFRA, EGFR* and *MYCN* (Lucas et al., 2021). Of most importance is that there has been little change in survival outcomes over the past 3 decades (Crowell et al., 2026). Successive studies using scRNAseq have further resolved our understanding of tumour cell states. DHG tumours, although arising in adolescents and young adults, have a molecular phenotype corresponding to interneuron progenitor cells which are found in the prenatal forebrain. Due to age of onset and stalled differentiation phenotype this leads to the consideration of DHG as a disease of brain development. Here, we review four key components of this disease and make a case that the transition to human pluripotent stem cell (hPSC)-derived 3D brain organoid models is important to address DHG and other rare, difficult to treat, diseases.

Firstly, we contextualise DHG in its neurodevelopmental mileu by giving an overview of corticogenesis from neural tube to postnatal forebrain. This is important to identify the developmental period that these tumours are recapitulating so that we can understand what biological processes are involved in driving corticogenesis, how cells move along the continuum of differentiation and how this can be implicated in developmental cancers. We then move on to briefly discuss epigenetics as the H3.3 histone variant is a critical epigenetic regulator. We discuss its role in regulating chromatin accessibility by post-translational modification (PTM) of specific residues on the histone tail and how this can result in epigenetic dysregulation in DHG. We link these two topics by a specific discussion on DHG as a disease of neural development and use this to segue to the current poor translational outcomes for new therapeutics and why this may be the case. We use this transition to introduce the hPSC-derived 3D brain organoid model. What follows is a discussion of this model’s emergence and its capacity to explore human disease mechanisms compared to other models, the current and future role of 3D brain organoid models in drug translation and the relevance for DHG which has seen little change in survival rates in decades.

There are many individual reviews that provide excellent resources for understanding epigenetics, corticogenesis and brain organoids, paediatric brain cancers and DHG specifically, and the emergence of new approach methodologies (NAMs). Although this is helpful for understanding these topics in depth, they are often siloed when addressing paediatric cancers. To understand the complexity of DHG, we traverse pillars of epigenetic dysregulation, neurodevelopment, accurate disease modelling and clinical translation. We review these four pillars to provide a holistic view of DHG from molecular and developmental underpinnings to translational opportunities. We contextualise why there has been little change in survival rates of patients with DHG, why DHG should be treated differently therapeutically and how we can achieve these goals utilising 3D brain organoids. We cannot truly understand this complex and devastating disease unless we look it as a whole; this is what we aim to achieve.

## From neural tube to postnatal forebrain development

### Neural tube specification and segmentation

Neurodevelopment is defined by the maturation and differentiation of neural progenitor cells (neural stem cells) into the mature cell types of the brain. During embryonic development, layers of cell types are added in waves that are orchestrated by morphogen gradients to determine brain region patterning and cell fate (Dessaud et al., 2008; Puelles et al., 2000). The gradients of specific morphogens are established along the dorsal to ventral, anterior to posterior, and rostral to caudal axes of the developing brain (Dessaud et al., 2008; Patten and Placzek, 2000).

Beginning from gastrulation, three distinct cell layers form, to which the anterior ectoderm (neuroectoderm) gives rise to a layer of differentiated neural precursor cells (NPCs) that are positioned along the rostral-caudal midline of the upper layer of the three-layered embryo (Stiles and Jernigan, 2010). This point of development is termed the neural plate. The formation of the neural tube arises from the folding or invagination of the neural plate, from human gestational week (GW) three, the fusion of two ridges of the neural plate forms a hollow tube with the anterior neuropore and posterior neuropore at either end (reviewed in (Wilson and Houart, 2004; Figure 1A). NPCs line the centre of the neural tube and as development continues, the hollow region will form the ventricular system of the central nervous system (CNS), with the NPCs lining the ventricular zone (VZ). As the caudal and anterior pores close, the process of segmentation is initiated by rapidly proliferating neural crest cells receiving signals from the notochord and mesoderm, and modules form distinct areas of the brain (Figure 1B). From gestational week 4, three distinct segments form, including the prosencephalon (forebrain), mesencephalon (midbrain) and rhombencephalon (hindbrain) (Puelles et al., 2013; Stiles and Jernigan, 2010; Figure 1B). The prosencephalon divides into the diencephalon and telencephalon, and the rhombencephalon into the metencephalon and myelencephalon, whereas the mesencephalon does not divide. This central structure gives rise to the entire nervous system and is the beginning of the distinct embryonic patterning that specifies the fate of these segments into the functional brain regions (Moore et al., 2020).

**FIGURE 1:**
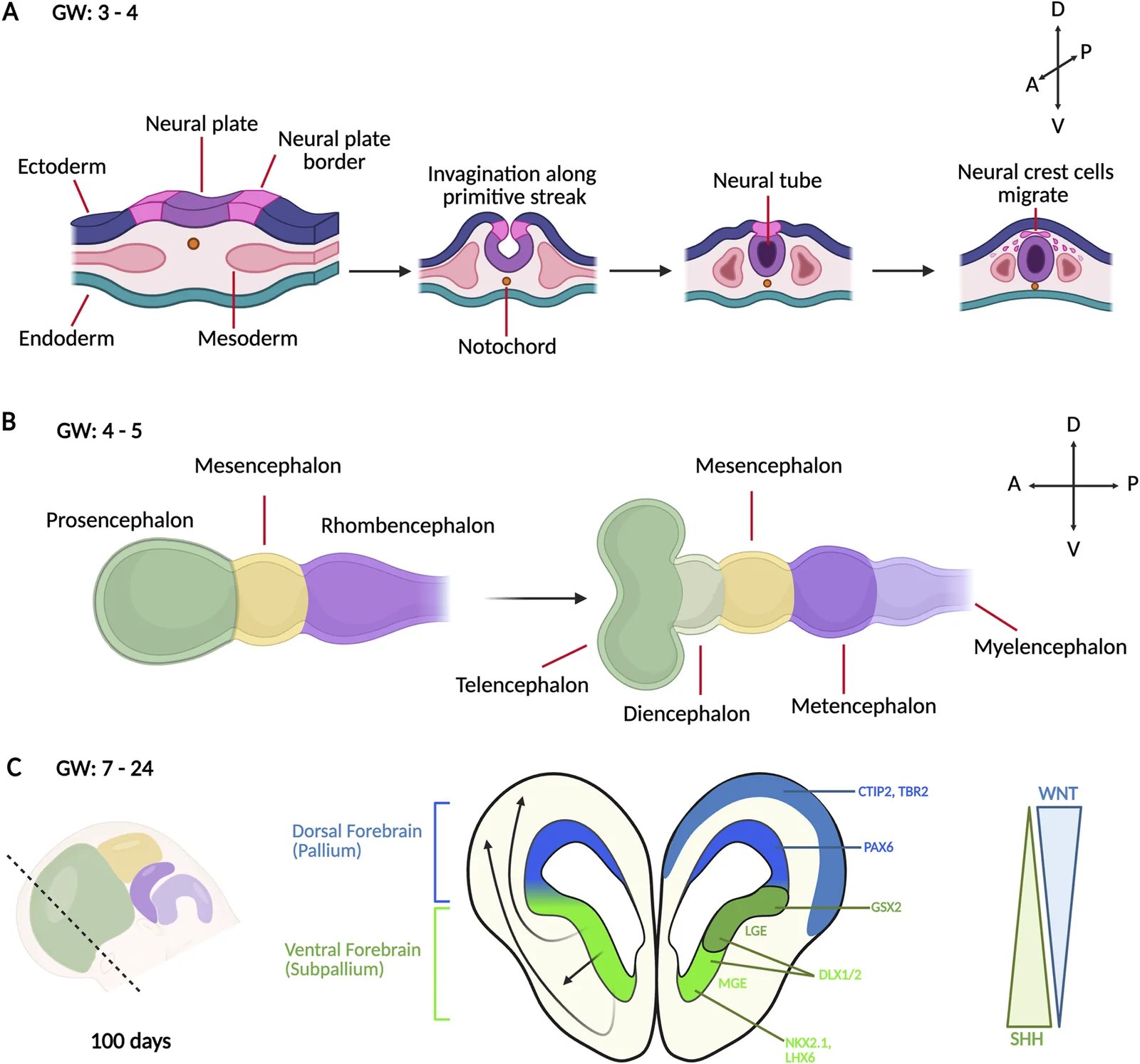
Human neurodevelopment from neural tube formation to embryonic forebrain development. **(A)** Formation of the neural tube from human gestational week (GW) 3–4. **(B)** Segmentation of the developing brain at GW4-5. Formation of the 3 distinct segments of the brain form (LHS) are then further divided into 5 major segments (RHS) between GW 4–5. **(C)** Forebrain development at GW 7–24. Developing brain structures at 100 days are shown with the same colour coding as in **(B)** indicating position of section. Coronal slice of the embryonic human forebrain at day 100 depicting the dorsal (Pallium) and ventral (Subpallial) forebrain. The transient structure of the human GE with key transcription factors and radial/tangential interneuron migration present from GW 7–24 are shown. WNT and SHH morphogen gradients are indicated. A = anterior, P = posterior, D = dorsal, V = ventral. MGE = medial ganglionic eminence, LGE = lateral ganglionic eminence. WNT = WNT signalling pathway activation, SHH = Sonic Hedgehog signalling pathway activation. Created in BioRender. Barry et al. (2026) https://BioRender.com/a768hce.

### Forebrain development and the cerebral cortex

Evolutionarily, the human cortex is one the most significantly distinct tissues from species such as the mouse and other primates (e.g., macaque). It is significantly enlarged compared to the mouse cortex, with a 1000-fold difference in the number of folds (Rakic, 2009). The human cortex’s protracted development continues well into early adulthood (Kelley and Paşca, 2022). Cortical neurogenesis begins in the VZ of the developing forebrain around GW seven and terminates between GW 20–24. The vast majority of all neurons produced in the human brain occurs during this period (Lewitus et al., 2014). This 112-day period of prolific development forms a large focus of this review and will be referred to throughout as the major cortical neurogenesis period (MCNP). Development of glial cell types begins towards the end of this proliferative period, at GW 20–24, and continues into early adulthood with oligodendrogenesis and myelination (Silbereis et al., 2016). Much of what we have learned about neurodevelopment has come from studies in mouse and chick and are largely conserved in human neurodevelopment (Shi et al., 2021; Molnár et al., 2019; Puelles et al., 2013). Indeed, neural stem cells (radial glia (RGC)) show broad conservation in transcriptional profiles during cortical neurogenesis, but specific signalling pathways, such as PDGF signalling, diverge in expression profiles in human compared to mouse (Lui et al., 2014). In this review, we focus on the developing forebrain and formation of the cortex by discussing seminal insights from studies in mouse and chick models to highlight the key mechanistic processes. We then contrast with the human brain to discuss the processes that are conserved in forebrain to cortex development until birth.

Once the prosencephalon divides into the telencephalon and the diencephalon, further subdivisions are referred to as prosomeres, the foundation of the Prosomeric Model, developed by John Rubenstein and Luis Puelles (Puelles and Rubenstein, 2003; Rubenstein et al., 1994; Marshall et al., 2023; Puelles et al., 2013) based on mouse and chick biology. The Prosomeric Model outlines the development of the embryonic brain from prosomeres in the forebrain and subsequent formation of distinct brain compartments depending on gene expression patterns and anatomical structure (Puelles and Rubenstein, 2003). The diencephalon develops into 3 prosomeres (p1, p2, p3), which are termed the pretectum, thalamus and pre-thalamus. The secondary prosencephalon, the telencephalon, develops into two hypothalamo-telencephalic prosomeres (hp1, hp2), later giving rise to the hypothalamus and telencephalon, respectively. The mesencephalon contributes to two prosomeres (m1, m2) (Puelles, 2018). The telencephalon is referred to as the developing forebrain and gives rise to the neocortex. The telencephalon can be divided into the pallium (dorsal forebrain) and subpallium (ventral forebrain) (Puelles et al., 2000) and is further arranged into the caudal ganglionic eminence (CGE), medial GE (MGE) and lateral GE (LGE) from ventral to dorsal along the subpallium (Figure 1C). Adjacent to the ventricles, are the VZ, sub-VZ (SVZ) and mantle zone (MZ). These three zones are arranged from most proximal to the ventricle to distally, respectively.

### Signalling and transcription factors in neurodevelopment

The process of differentiation of NPCs in the developing mammalian embryonic forebrain is orchestrated by a host of cell signalling pathways and transcription factors (Silbereis et al., 2016). The pallial/subpallial gradient is defined by Wnt and Sonic hedgehog (SHH) signalling, where Wnt activation specifies a dorsal fate and SHH activation specifies ventral fate in a gradient fashion (Figure 1C). Key transcription factors also define these regions, such as dorsally expressed PAX6 and TBR2, whereas DLX2 and NKX2.1 establish the ventral forebrain (Georgala et al., 2011; Lindtner et al., 2019; Du et al., 2008; Puelles et al., 2000). The differential expression of transcription factors defines the pallial/subpallial boundary where dorsally expressed PAX6 and ventral DLX2 expression form a boundary at the dorsal LGE (Figure 1C). In mice, when *Pax6* is knocked out, the ventral forebrain expands and conversely, there is expansion of the dorsal forebrain upon loss of *Dlx2* (Kroll and O'Leary, 2005; Piñon et al., 2008).

Key signalling pathways (e.g., Wnt and SHH) and transcription factors (e.g., PAX6 and DLX2) control the prolific production of new neurons during the MCNP in humans. In particular, the curation of signalling and gene regulatory networks leads to the two main distinct lineages of neurons, glutamatergic excitatory neurons and GABAergic inhibitory interneurons, that are derived from the developing forebrain and populate the neocortex to provide a balance of excitatory and inhibitory neural activity.

### GABAergic inhibitory interneuron development

The GE of the ventral forebrain in mice is a source for newly born GABAergic interneurons which migrate dorsally, mature, and populate the developing neocortex as inhibitory neurons (Marín and Rubenstein, 2001). This is also true in humans, however, there are subtle and key distinctions: in mice, two-thirds of inhibitory neurons arise from the ventral and one-third from the dorsal forebrain; however, the inverse proportions are postulated in the developing human brain (Anderson et al., 1997; Letinic et al., 2002). The GABAergic interneuron lineage in the mouse is regulated by transcription factors such as ASCL1, GSX2, DLX1, -2, -5 and -6. These transcription factors are expressed in the GE of the developing forebrain and are required to promote GABAergic interneuron specification and tangential migration from the ventral forebrain to the dorsal neocortex (Andersen et al., 2020; Marín and Rubenstein, 2001; Rubenstein et al., 2024; Toresson and Campbell, 2001), and directly repress genes required for oligodendrocyte formation (Jiang et al., 2020; Leung et al., 2026; Petryniak et al., 2007; Chapman et al., 2018; Chapman et al., 2013; Pei et al., 2011; Waclaw et al., 2009; Yun et al., 2003; Leung et al., 2026).

There are many subtypes of GABAergic interneurons that develop from the human GE, and almost all are conserved with rodents; however, at least two populations of human interneurons have been identified that are not shared with rodents: a prospective subtype of MGE-derived fast-spiking interneuron and a large population of CGE-derived GABAergic interneurons (Shi et al., 2021). Access to human brain tissue and the development of human transcriptomic atlases has recently revealed even more subtypes. For example, in the developing forebrain there is evidence of a novel GABAergic neuronal lineage, where the migration of CRABP-expressing cells from the medial ganglionic eminence result in specific parvalbumin (PVALB) neurons in the thalamus (Braun et al., 2023).

### Glutamatergic excitatory neuron development

Conversely, the glutamatergic excitatory neuronal lineage arises primarily in the developing dorsal forebrain (pallium). This region is defined by expression of transcription factors including PAX6, EMX2, TBR2 and CTIP2 (Figure 1C) in both mouse and human (Puelles and Rubenstein, 2003; Hansen et al., 2013). First identified in mice and later found to be conserved in human, newborn excitatory neurons are born in the VZ of the pallium during the MCNP from asymmetrically dividing RGCs which differentiate to intermediate progenitors and then excitatory neurons (Hodge et al., 2019). These neurons migrate radially to the cortical plate where the 6 distinct layers of the cortex form and present with vesicular glutamate transporters (vGLUT) that regulate transportation of the neurotransmitter glutamine. The large majority of these are pyramidal neurons which are found in layers II-IV and are characterised by CUX1/2 and SATB2 (Planert et al., 2026). Different subtypes of pyramidal neurons occupy different layers and when fully developed, project not only throughout the cortex but to the thalamus, striatum, motor cortex and brainstem (Planert et al., 2026). This layering is a crucial feature specific to human brain development, where layers II-III are much expanded in comparison to the mouse (Planert et al., 2026). Human brain atlases have again proven to be important in resolving lineage specificity in human cortex development. For example, truncated RGCs maintain a glutamatergic neurogenic potential for a protracted period (Keefe et al., 2025). Furthermore, glutamatergic neurons born later in gestation from truncated RGCs form the deep cortical layer neurons and may contribute to the expansion of the subplate region during mid-gestation (Keefe et al., 2025).

The proliferation and differentiation of glutamatergic excitatory neurons and GABAergic inhibitory interneurons is at its most prolific during the MCNP. At the end of the MCNP, the upper cortical layers are (II-IV) are still forming and following this period there is continued migration and proliferation until birth; however, both are greatly decreased after the MCNP. The period until birth is characterised by maturation of neurons through massive elongation of axons and dendrites, synaptogenesis, the defining of specific cortical areas, continual depolarisation of the intracellular environment for GABAergic neurons to become inhibitory, and developing spontaneous neural circuitry (Nowakowski et al., 2017; Silbereis et al., 2016). Subsequently, after birth synaptic pruning mediated through the complement system occurs as cortical neurons mature but do not proliferate (De Felipe et al., 1997).

### Species differences in cortex development

Although these processes are highly conserved from mouse to primate to human, a number of studies have identified key differences that could add to the diversity of neurons and the difference in cortical structure. These include the identification of two new subpallial derived inhibitory neuron classes mentioned above, in addition to at least 10 different subtypes of rosehip neurons found in cortical layer I that are derived from the GABAergic lineage (Boldog et al., 2018; Hodge et al., 2019; Shi et al., 2021). Indeed, even RGCs, although primarily conserved between species, show distinct differences in specific signalling pathways such as PDGFD- PDGFRβ with high expression in the human dorsal forebrain but not in the mouse (Lui et al., 2014). We have provided a glimpse of corticogenesis from development of the neural tube to birth with a particular focus on transcription factors and signalling pathways that define the developing forebrain and the two main cell lineages that arise during the MCNP. While current understanding stems from studies in mice and chick, defining the differences in human brain development is critically important for appreciating the greater complexity of the human cortex.

This discussion on corticogenesis describes the neurodevelopmental dynamics at play during the human gestational period. In DHG, most of these processes are important to address how cells in the postnatal human tumour recapitulate this developmental epoch. The molecular phenotype of DHG is most akin to early interneuron progenitors, those which are born in the developing forebrain and migrate to the neocortex (Liu et al., 2024a). Key homeobox gene encoded transcription factors, such as DLX2 and GSX2, are important for this process in normal development, but appear to be dysregulated in DHG, as an example (Chen et al., 2020). We highlight the differences between human and mouse as model systems, in part because the human and mouse brains are very different. Do small changes in neurodevelopment that are conserved or not conserved impact or inform our ability to better understand DHG? The other side to this, which we will discuss in the next section, is how epigenetics plays a significant role. H3.3 mutations can affect chromatin accessibility, and this is important in both neurodevelopmental and tumorigenic contexts.

## Epigenetics and mammalian brain development

In addition to the tightly regulated morphogen gradients and transcription factors described above, epigenetic regulation of chromatin accessibility plays a major role in governing neurodevelopment. The precise opening and closing of chromatin in specific regional and temporal waves provide a key mechanism for orchestrating neural development from stem cell to mature cortex.

### Regulation of chromatin accessibility by modification of DNA and post-translational modification of histones

The deposition and combination of methylation, acetylation and phosphorylation of DNA or histones regulate the opening and closing of chromatin and dictate protein expression. DNA methylation of cytosine nucleotides at the C^5^ position directly regulates the activation of gene promoter regions (Egger et al., 2004). Methylation of DNA is maintained by DNA methyltransferases (DNMT) 3A and 3B. In most cases, the cytosine must be followed by a guanine to be methylated (CpG). Around 70% of CpG dinucleotides are methylated, while the rest are clustered together, predominately in intergenic regions and transcription start sites called CpG islands (Cooper and Krawczak, 1989; Illingworth et al., 2008). CpG islands located within promoter regions are almost always unmethylated as transcription factors block DNMT access. It allows for transcription complexes to bind and activate gene expression. Methylating CpG islands can induce a local structural rearrangement to silence gene expression by blocking transcription factor binding (Day and Sweatt, 2010).

A second key component of epigenetic regulation involves post-translational modification (PTM) of histone proteins. The four core histones that make up the nucleosome (H2A, H2B, H3 and H4) are replicative and synthesized during S-phase to fill voids following chromatin replication (Egli et al., 2008; Wong and Tremethick, 2025). Non-replicative histones are actively inserted into the chromatin to regulate chromatin accessibility. For example, H3.3 is a multifunctional non-replicative histone variant of H3 that is usually inserted into chromatin to actively replace H3 (Tagami et al., 2004). The insertion of non-replicative histone variants occurs outside of S-phase and is a dynamic process that can alter the structure of the chromatin and gene expression (Venkatesh and Workman, 2015). H3.3 is encoded by *H3-3A* (formerly *H3F3A)* and is expressed throughout the cell cycle (except during mitosis). H3.3 is inserted into the euchromatin or heterochromatin by two chaperone systems, the HIRA-UBN1-CABIN1 (HIRA) or the Alpha-Thalassemia/mental Retardation X-linked (ATRX) and Death Domain Associated Protein (DAXX) complex (referred to as DAXX) (Gatto et al., 2022; Michod et al., 2012; Ray-Gallet et al., 2002). These chaperones specifically recognise the four amino acids A^87^AIG^90^ within H3.3 that are distinct from the H3 histone (Figure 2A). The HIRA complex is recruited to insert H3.3 in nucleosome depleted regions, particularly during gene transcription (Gatto et al., 2022; Ray-Gallet et al., 2002), whereas the DAXX complex adds H3.3 proteins at repetitive heterochromatic regions such as pericentromeric and telomere regions, in addition to active regulatory elements (Michod et al., 2012; Navarro et al., 2020). However, it is unknown what factors determine which chaperone system is used (Cohen and Meshorer, 2024).

**FIGURE 2:**
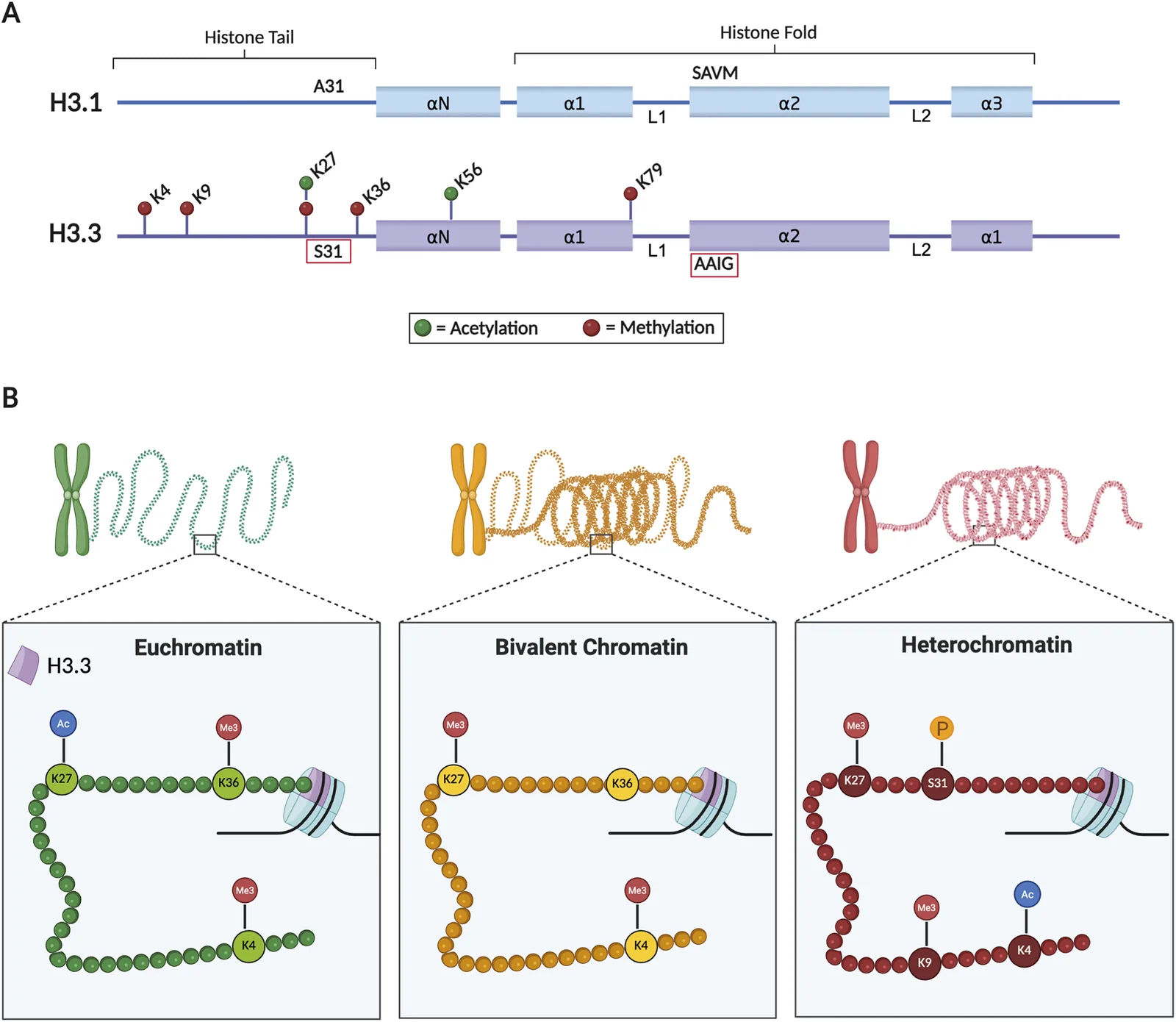
Post-translational modification of non-replicative histone variant H3.3. **(A)** Schematic representation of the structure of the replicative H3.1 histone and the non-replicative H3.3 histone variant. The N-terminal histone tail (40 residues) and histone fold of the 147 aa H3 peptide are indicated. Red boxes indicate amino acid changes in H3.3 compared to H3.1. Lysine (K) and Serine (S) residues that are post-translationally modified are highlighted. **(B)** Schematic showing alternative PTMs on the H3.3 tail for different chromatin accessibility states: Euchromatin is open, bivalent chromatin is poised and heterochromatin is closed. Created in BioRender. Barry et al. (2026) https://BioRender.com/h5c9ia3.

H3.3 is distinct from other histones through the addition of PTMs to key residues within the histone tail that actively regulate chromatin dynamics (Wong and Tremethick, 2025). The reversible deposition and removal of methyl and acetyl groups to the highly conserved lysine residues within histone tails is dynamic and can alter cell fate by expression of downstream proteins (Wong and Tremethick, 2025). The methylation or acetylation states present at multiple locations on the H3.3 tail play specific roles in chromatin accessibility and gene activation (Cohen and Meshorer, 2024). At the embryonic stage, tri-methylation at H3K27 (H3K27me3) represses genes and stabilises heterochromatin, particularly around the pericentromeric regions. H3K4me3 and H3K9ac mark active promoters whereas H3K27ac marks active enhancers and H3K36me3, gene bodies of active genes (Millán-Zambrano et al., 2022; Figure 2B). On the same histone tail, H3K27me3 and H3K4me3 can coexist in a transient state to which genes are poised to be on or off, a state referred to as bivalent chromatin (Bernstein et al., 2006; Figure 2B). PTMs at these specific residues designate this region as facultative heterochromatin, in that the heterochromatin is dynamic, depending on insertion of H3.3 and the relative PTMs.

A more specific example of how PTMs work in a combinatorial fashion is how H3.3 maintains active euchromatin. On the H3.3 tail, K4me3, K27ac, and K36me3 maintain active regions of chromatin whereby K4- and K36- tri-methylation allosterically inhibit the polycomb repressor complex 2 (PRC2), thereby preventing methylation at K27 which propagates silencing of chromatin regions (Schmitges et al., 2011). The role of H3.3 and the active and repressive marks is essential in early development. Studies in mouse models have been instructive in determining mechanisms of regulation. *H3-3A* knockout in mice results in embryonic lethality due to the essential role for H3.3 in establishing heterochromatin and gene repression (Jang et al., 2015; Smith et al., 2022). The introduction of point mutations at K27 that prevent either methylation or acetylation in H3.3 leads to developmental arrest as early as the two-cell stage due to impaired heterochromatin formation which triggers mitotic failure, chromatin aberrations and embryonic lethality, underscoring the finely tuned state of H3.3 modifications required for embryonic development (Santenard et al., 2010). In comparison, point mutations at K27 in *H3C2* (formerly *HIST1H3A*) which encodes the replicative Histone 3.1, do not lead to developmental arrest, highlighting the importance of H3.3 in early development.

### Histone modifying enzymes alter methylation and acetylation states to regulate neuronal development

Given the importance of epigenetic regulation during development, several DNA and histone modifying enzymes have been shown to be implicated in neural development and disease (Table 1). These methyl and/or acetyl modifiers are also not restricted to modifying histone proteins but have broader functions, which is reflected in changed nomenclature (Narita et al., 2019). For example, general control non-depressible 5 (GCN5), previously defined as a histone acetyltransferase (HAT), is now referred to as lysine acetyltransferase 2 A (KAT2A). KAT2A is required for neural tube closure (Bu et al., 2007; Xiao et al., 2023). Lysine methyltransferase 6 (KMT6), an histone methyltransferase (HMT) enzyme enhancer of zeste homolog 2 (EZH2), a catalytic subunit of the polycomb repressive complex-2 (PRC2), is suggested to control neural progenitor state and prevent premature neural differentiation through depositing methyl groups on the H3.3 K27 residue (Pereira et al., 2010; Rai et al., 2010; Rai et al., 2010; Pereira et al., 2010). It is also implicated in the mechanism for diffuse midline glioma (DMG, formerly DIPG) formation, where the H3K27M driver mutation prevents PRC2 methylation of the K27 residue. The resulting global hypomethylation results in dysregulated chromatin arrangement possibly contributing to a stalled differentiation phenotype where the tumour cell of origin is stuck as an oligodendroglial progenitor cell (OPC)-like cell (Khuong-Quang et al., 2012; Liu et al., 2022; Schwartzentruber et al., 2012). In addition, DNA methyl transferases (DNMT) such as DNMT3a, has been shown to be required for neuromuscular junction and synaptic function in forebrain neurons, and its depletion can cause neurodegeneration (Feng et al., 2010; Nguyen et al., 2007; Khazaei et al., 2023; Table 1).

**TABLE 1:** Epigenetic modifier enzymes in neurodevelopment.

Enzyme	Functional class and name	Overarching function	Effect on neurodevelopment & disease
HAT1 (Histone acetyltransferase 1)	KAT1 Type B lysine acetyltransferase localised primarily in the cytoplasm (Gcn5-related N-acetyltransferase (GNAT) family) (Ortega et al., 2023)	Acetylates proteins including histones, mitochondrial proteins and transcription factors (Agudelo Garcia et al., 2020) Acetylates K5 and K12 on nascent H4 within the cytoplasm facilitating chromatin structural assembly (Nagarajan et al., 2013; Sobel et al., 1995)	Deficiency leads to embryonic lethality, skull and bone structural defects and impaired cell proliferation (Nagarajan et al., 2013)
GCN5 (Gcn5-related N-acetyltransferase)	KAT2A Type A lysine acetyltransferase localised primarily in the nucleus (GNAT family) (Brownell et al., 1996)	Acetylates histones and acetylates and/or modulates multiplex transcription complexes (Georgakopoulos et al., 1995; Marcus et al., 1994) Acetylates lysine residues on H2B, and K9, 14 and 18 on H3 to activate chromatin (Allis et al., 2007)	Deficiency leads to embryonic lethality, dysfunctional apoptosis and neural tube closure defects (Bu et al., 2007; Xu et al., 2000)
PCAF (P300/CBP-associated factor)	KAT2B Type A lysine acetyltransferase (GNAT family) (Herrera et al., 1997)	Acetylates proteins including histones and cell cycle regulating transcription factors (Love et al., 2012; Herrera et al., 1997) Deposits acetyl groups to K9, 14 and 18 on H3 to activate chromatin (Allis et al., 2007)	Deficiency leads to impaired memory and stress response (Maurice et al., 2008), but does not lead to embryonic lethality due to compensatory GCN5 upregulation (Yamauchi et al., 2000)
CBP (CREB-binding protein)	KAT3A Type A lysine acetyltransferase (P300/CBP family)	Transcriptional coactivator which acetylates proteins including histones and transcription factors (Kikuchi et al., 2023) Deposits acetyl groups to K5 on H2A, K12 and K15 on H2B and N-terminal K on H3/H4 (Allis et al., 2007; Kikuchi et al., 2023)	Deficiency linked to Rubinstein-Taiby syndrome (Negri et al., 2015; Tsai et al., 2011), 16p13.3 duplication syndrome (Thienpont et al., 2010), embryonic lethality (Yao et al., 1998), interneuron differentiation (Tsui et al., 2014) memory and cognitive deficits (Viosca et al., 2010)
P300 (E1A-associated protein p300)	KAT3B Type A lysine acetyltransferase (P300/CBP family)	Transcriptional coactivator which acetylates proteins including histones and transcription factors (Jin et al., 2010) Acetylates K5 on H2A, K12 and K15 on H2B and N-terminal lysine residues on H3/H4 (Allis et al., 2007; Liu et al., 2008b)	Deficiency linked to Rubinstein-Taiby syndrome (Negri et al., 2015; Tsai et al., 2011), embryonic lethality (Yao et al., 1998), memory and cognitive deficits (Viosca et al., 2010)
HDAC1 (Histone deacetylase 1)	KDAC family Class I Zinc-dependent lysine deacetyltransferase (Taunton et al., 1996)	Forms co-repressor complexes to deacetylase and/or modulate proteins including histones, nonhistone transcription factors and cell cycle regulators (Alland et al., 2002; Lagger et al., 2002; Zhou et al., 2018)	Deficiency leads to embryonic lethality (Montgomery et al., 2009), cell cycle dysfunction, and impaired neural differentiation (Montgomery et al., 2009; Nieto-Estevez et al., 2021)
HDAC5 (Histone deacetylase 5)	KDAC family Class IIa Zinc-dependent lysine deacetyltransferase (Grozinger et al., 1999)	Deacetylates lysine residues on histones, deacetylates and/or modulates nonhistone transcription factors and chaperone protein 14-3-3 (Fischle et al., 2002; Grozinger et al., 1999)	Deficiency leads to compromised axonal regeneration, impaired neural differentiation (Schneider et al., 2008), modulate neuropathic pain pathways (Westlund et al., 2024) and behavioural deficits (Barry et al., 2026)
SIRT1 (Sirtuin 1)	KDAC family Class III NAD + dependent lysine deacetyltransferase (Vaquero et al., 2004)	Deacetylates primarily K16 on H4 and K9 on H3, deacetylates and/or regulates nonhistone transcription factors, modulates oxidative stress and exerts metabolic effects (Huang et al., 2021; Liang et al., 2020; Qiang et al., 2011; Rada et al., 2018; Vaquero et al., 2004)	Deficiency leads to impaired synaptic plasticity and neurogenesis (Hisahara et al., 2008; Michán et al., 2010), disrupted neuronal differentiation (Hisahara et al., 2008) and dysfunctional autophagy (Liang et al., 2020)
HDAC11 (Histone deacetylase 11)	KDAC family Class IV Zinc-dependent lysine deacetyltransferase (Gao et al., 2002)	Forms transcriptional complexes with other HDACs (Joshi et al., 2013), conducts defattyacylase activity (Kutil et al., 2018) and to a minimal extent deacetylates lysine residues on histones (Cao et al., 2019)	Deficiency can lead to disruption to oligodendrocyte and neuronal maturation (Liu et al., 2008a) and dysfunction of the motor neuron complex (Joshi et al., 2013)
MLL (Mixed-Lineage Leukemia 1)	KMT2A Lysine methyltransferase	Catalyses mono-, di- and tri- methylation of K4 on H3 to mark chromatin as transcriptionally active (Guenther et al., 2005) Regulates transcription of Hox genes and other transcription factors to maintain epigenetic memory and development (Ernst et al., 2004)	Deficiency leads to disrupted cell differentiation programs, upregulated Hox gene expression contributing to oncogenic properties (Xu et al., 2016)
SETD2 (SET domain containing 2)	KMT3A Lysine methyltransferase	Trimethylates K36 on H3 to actively repress chromatin and transcription (Sun et al., 2005; Edmunds et al., 2007) Forms complexes with DNA and other proteins to regulate nucleosome functional dynamics (Carvalho et al., 2013)	Deficiency leads to disrupted differentiation programs of embryonic stem cells in the endoderm lineage, Luscan-Lumish syndrome (Zhang et al., 2014; Hu et al., 2010)
EZH2 (Enhancer of Zeste Homolog 2)	KMT6 Lysine methyltransferase	Subunit of the Polycomb repressive complex 2, essential for trimethylation of K27 on H3 (Højfeldt et al., 2018) and can bind to a variety of transcription factors (Shi et al., 2007; Lee et al., 2011)	Deficiency leads to embryonic lethality (O'Carroll et al., 2001), impaired pluripotency in embryonic stem cell development (Shen et al., 2008) and stalled cell fate (Liu et al., 2022)
DNMT1 (DNA methyltransferase 1)	Cytosine-5 DNA methyltransferase	Transfers methyl groups to CpG sites on genomic DNA and histones, conserving epigenetic memory (Bacolla et al., 1999; Pedrali-Noy and Weissbach, 1986) and forms complexes with various transcription factors (Kim et al., 2002; Rountree et al., 2000; Viré et al., 2006) to support stable gene expression	Deficiency leads to embryonic lethality (Li et al., 1992), reduced neuronal dendrite growth (Hutnick et al., 2009), gene imprinting defects (Kaneda et al., 2004; Bourc’his et al., 2001) and limited cell viability (Liao et al., 2015)
DNMT3A (DNA methyltransferase 3A)	Cytosine-5 DNA methyltransferase	Conducts *de novo* methylation of CpG sites on DNA to preserve methylation patterns during neurodevelopment (Chen et al., 2002)	Deficiency leads to embryonic lethality (Okano et al., 1999), gene imprinting defects (Kaneda et al., 2004; Bourc’his et al., 2001; Hata et al., 2002) and age-related cognitive decline (Oliveira et al., 2012)
TET1 (Ten-Eleven Translocation 1)	Fe(II)/α-ketoglutarate-dependent dioxygenase DNA demethylase	Converts 5-methyl cytosine to 5-hydroxyl-methyl-cytosine in DNA to transcriptionally activate chromatin (Tahiliani et al., 2009; Ito et al., 2010; Zhu et al., 2016)	Deficiency leads to impaired neural progenitor cell proliferation, reduced synaptic plasticity, impaired memory and cognition (Zhang et al., 2013; Rudenko et al., 2013; Hutnick et al., 2009)
LSD1 (Lysine specific histone demethylase 1)	KDM1A Lysine demethylase	Demethylates mono- and di- methyl K4 marks on H3 to repress transcription programs (Forneris et al., 2005; Shi et al., 2004)	Deficiency leads to disrupted embryonic development, impaired stem cell differentiation programs and intellectual disability (Foster et al., 2010; Del Blanco et al., 2024)
UTX (Ubiquitously transcribed tetratricopeptide repeat X-linked protein)	KDM6A Lysine demethylase	Demethylates di- and tri- methyl K27 marks on H3 to de-repress chromatin and activate transcription (Hong et al., 2007)	Deficiency leads to Kabuki syndrome (Van Laarhoven et al., 2015) and deficits in synaptic plasticity and age/sex-related cognitive effects (Shaw et al., 2023)

The use of histone modifying and DNMT inhibitors has been explored for treatment of neurodevelopmental disorders. However, these enzymes function in a range of cell types and tissues. DNMTs are important *de novo* methylators, meaning from fertilization, regulating gene expression by methylating CpG dinucleotides to orchestrate development of the human embryo into adulthood (Edwards et al., 2017). Lysine methyltransferases (KMTs) are predicted to methylate over a thousand different proteins and there are over a hundred different types of KMTs that operate in all tissues of the body (Husmann and Gozani, 2019). Targeted treatments for epigenetic disorders is therefore challenging. Small molecules that aim to target epigenetic regulators have varying success because targeting one epigenetic modifier enzyme may be favourable *in vitro*, yet *in vivo* application causes toxicity preventing clinical translation. A recent example is the use of a pan-HDAC inhibitor Panobinostat for treatment of DMG, which showed significant effects on cell differentiation *in vitro* and was further validated in mice *in vivo* (Lin et al., 2019). However, a more recent study has shown major toxicity in the form of myelosuppression with no observed clinical benefits for patients (Monje et al., 2023).

### Epigenetic disorders, complications and treatments

As gene expression is linked inextricably with epigenetic regulation, it follows that any epigenetic dysregulation would likely perturb developmental trajectories and cause serious medical consequences. There are multiple examples of dysregulation of epigenetic modifying enzymes that induce aberrant neurological function (Table 1). Facioscapulohumeral dystrophy (FSHD) is an epigenetically dysregulated disease resulting in weakness of facial, shoulder and upper arm muscles. It is caused by hypomethylation of D4Z4 microsatellite repeats on chromosome 4q35. Hypomethylation at D4Z4 results in loss of epigenetic repression of the transcription factor Double homeobox 4 (DUX4) which causes muscle toxicity and degeneration (Daxinger et al., 2015). Similarly, Angelman Syndrome (AS) is a neurodevelopmental disorder that also results from aberrant epigenetic regulation. AS is characterised by severe mental retardation, ataxia, seizures, EEG abnormalities and bouts of inappropriate laughter (Lalande and Calciano, 2007). In AS, mutations in the maternal allele of UBE3A impair the E3 ubiquitin ligase function in mature neurons. The paternally inherited copy of UBE3A is epigenetically silenced by long non-coding antisense RNA so that only the maternal UBE3A is normally expressed in neurons, but in AS maternal allele mutations result in insufficient levels of UBE3A protein. Recent studies aim to reactivate the paternal UBE3A to overcome the loss of UBE3A protein (Lalande and Calciano, 2007; Liu et al., 2024b; Vihma et al., 2024). In these instances, epigenetic dysregulation is linked to disease phenotypes that have devastating neurological effects. These examples also exhibit how mechanistically wide-ranging epigenetic disorders are, which adds great complexity to patient treatments. To grasp this complexity, it will be important to explore the multiple facets of epigenetic regulation in specific instances of neurodevelopmental epigenetic disorders.

We have provided an overview of the epigenetic regulation of chromatin by DNA methylation and histone modification, with a particular focus on the histone variant H3.3. We have discussed how residue-specific PTMs on the histone tail have a flow on effect for DNA methylation and deposition/removal of acetyl and methyl groups on other residues through interactions with epigenetic modifiers. We have further provided details on chromatin accessibility and how it is implicated in disease to the effect that H3.3 mutations play a role in disease aetiology. In the following section, we speak directly to DHG and use the epigenetic and neurodevelopmental contexts provided in earlier sections to guide our discussion regarding the complexity and underlying mechanisms of this disease.

## Paediatric high-grade gliomas and H3.3 G34 mutant diffuse hemispheric glioma (DHG) - an example of an epigenetic neurodevelopmental disease

The paediatric high-grade gliomas (pHGG) are diseases molecularly defined by epigenetic dysregulation (Filbin and Monje, 2019). Here we explore the epigenetic mechanisms that drive pHGG as well as current treatment and novel therapies. CNS tumours are the leading cause of cancer related deaths in children (Ostrom et al., 2020) of which pHGGs include some of the most aggressive subtypes and are classified by the World Health Organisation as grade 4 tumours (Bavle and Chintagumpala, 2018). More than 30% of pHGGs are classified as DHG, H3.3 G34 mutant, and arise in the cerebral hemispheres of adolescents and young adults (AYA) (Buccoliero et al., 2022). DHG is molecularly defined by a heterozygous mutation in the *H3-3A* (formerly *H3F3A)* gene which encodes the histone variant H3.3, resulting in the substitution of a glycine for either an arginine or valine at residue 34 (Schwartzentruber et al., 2012). 90% of DHG harbour the arginine mutation, and the remaining 10% contain valine at this position (Vuong et al., 2022). DHG onset ranges from 13–22 years old and the median survival rate ranges from 15–22 months post-treatment (Crowell et al., 2022). The driver mutation in DHG occurs in *H3-3A*/*H3F3A* and results in expression of the H3.3 G34R/V, often accompanied by co-mutations such as loss of *TP53*, *ATRX* (over 90% of cases) and amplification of *PDGFRA, EGFR* and *MYCN* (Lucas et al., 2021).

The current standard of care for DHG is maximal safe surgical resection followed by chemoradiation (60 Gy in 30 fractions over 6 weeks with concurrent daily temozolomide) followed by adjuvant temozolomide (5 days per 28 day cycle) for 6–12 4 week cycles (Erker et al., 2024). There are no supportive Phase 3 randomised clinical trial data for pHGG including DHG. Preferably, the current standard of care is to enrol the patient on a clinical trial for novel adjuvant therapies such as radiation sensitizers or targeted therapies if there is a concomitant actionable mutation such as EGFR and PDGFR. Targeted therapies include CAR T-cell/immunotherapies and epigenetic therapies such as the BET inhibitor JQ1 (Day et al., 2022; Sweha et al., 2021; Vanan et al., 2017). There are no targeted therapies currently approved for treatment to include in standard of care due to low efficacy or toxicity observed in the clinic. Hence, there is much to be done for discovery research to improve treatments.

### Neurodevelopmental background of DHG–tumour cells recapitulate embryonic forebrain signatures

Access to paediatric samples has improved over the last decade due to data proving safety of biopsies along with the quality of data extracted from biopsies due to next-generation sequencing technologies influencing family engagement (Wang et al., 2015). This has allowed molecular analysis of DHG tumours which delineate these tumours from adult glioma by both key driver mutations and their developmental timepoint. DHG is a heterogenous tumour with neuronal and glial-like compartments, yet investigation through single cell sequencing has defined its molecular profile to closely resemble a GABAergic interneuron lineage and shows that DHG tumours recapitulate a more progenitor-like interneuron state along the differentiation continuum (Chen et al., 2020; Liu et al., 2024a). This is reflected by patient samples exhibiting expression patterns of canonical interneuron and ventral forebrain markers including transcription factors such as DLX2 and GSX2, and loss of glial progenitor markers such as OLIG2. Even within this discovery, there is still much to be understood, for example, whether the GABAergic interneuron progenitor lineage of this tumour is derived from the medial, lateral or caudal ganglionic eminences (MGE, LGE and CGE, respectively) of the ventral forebrain. Alternatively, are DHG cells derived from a dorsal, cortical interneuron progenitor pool (Letinic et al., 2002)? In Liu et al. (2024a) it is purported that the GABAergic interneuron precursors are ganglionic eminence-like; however, the authors state that these cells exhibit gene signatures from all 3 GE structures. In this data set, they compare human tumour cells with mouse ganglionic eminence scRNAseq data to assist with this analysis. However, this dataset may affect the current determination of where these cells are migrating from and what cell types they most closely recapitulate in the developing human forebrain. As access to human prenatal tissues becomes more available, and bioinformatic tools become more refined, answering these questions will become achievable.

Despite recent single cell sequencing data, DHG is still a relatively understudied tumour and there is still a lot to understand about the broader developmental mechanisms. The underlying stall in differentiation and prenatal signature of the developing forebrain in DHG tumours is thought to result from the epigenetic H3.3 G34-mutation (Schwartzentruber et al., 2012; Tlais et al., 2026). Even though the onset of DHG occurs in adolescence and young adulthood, H3.3 G34R/V mutations may be acquired during cortex development, particularly as the molecular characteristics of DHG resemble interneuron precursors (Chen et al., 2020; Liu et al., 2024a). Alternatively, G34-mutant cells may revert to an early developmental stage and de-differentiate as mutations arise. In any case, DHG tumours are now considered as a disease of development through epigenetic dysregulation (Filbin and Monje, 2019).

### DHG: an epigenetic disorder

As described above, the N-terminal histone tail is crucial in maintenance of chromatin accessibility. There are a number of mechanisms proposed to explain how the H3.3 G34R/V mutation causes gene expression changes and DHG mutant phenotype, all of which stem from steric inhibition from the bulky Arg or Val substituted residue preventing local epigenetic modifier regulation (Khazaei et al., 2023; Schmitges et al., 2011; Huang et al., 2020; Jain et al., 2020). The impact of G34R/V is understood to affect the neighbouring K36 residue within the histone tail. K36 is well characterised as a residue that is crucial for chromatin remodelling (Li et al., 2019). The H3.3 G34R/V mutation is proposed to allosterically block the ability of set domain containing 2 (SETD2), a lysine methyltransferase, to deposit methyl groups on residue K36 resulting in genomic instability (Schmitges et al., 2011; Figure 3A). This leads to global epigenetic changes by allowing PRC2 to access chromatin, recruit Enhancer of Zeste 1 PRC subunit (EZH1) to silence genes, creating sweeping repressive effects (Chen et al., 2020; Huang et al., 2020; Jain et al., 2020; Schmitges et al., 2011; Figure 3A). Similarly, DNA Methyltransferase 3A (DNMT3A) is proposed to be implicated in the H3.3 G34R dysregulation. DNMT3A acts by its Pro-Tryp-Tryp-Pro (PWWP) and ATRX-DNMT3-DNMT3L (ADD) domains to bind unmodified K4 and methylated K36 residues (Kubo et al., 2024; Qiu et al., 2002). DNMT3A binds preferentially to nucleosomes, then it methylates the linker DNA flanking this nucleosome (Kelly et al., 2012; Xu et al., 2020). When access to K36 is allosterically inhibited by G34 mutations, it impairs the function of DNMT3A so it cannot deposit methyl groups on the target DNA and hence transcription is erroneously kept active (Figure 3B). The loss of methylation to K36 residue links the SETD2 and the DNMT3A mechanisms. These two mechanisms can form a lethal combination, where interneuron progenitor and stem genes are left open, yet lineage maturing regions are closed prematurely. Many of these studies have relied heavily on mouse biology for mechanistic validation. Although some core mechanism may be conserved, it’s unique effects in a human context may produce additional findings. This may explain why there has been no successful translation of therapeutic approaches. The interplay between neurodevelopment, epigenetic mechanism and cell fate necessitates a system that can capture the complexities of both human brain and tumour development.

**FIGURE 3:**
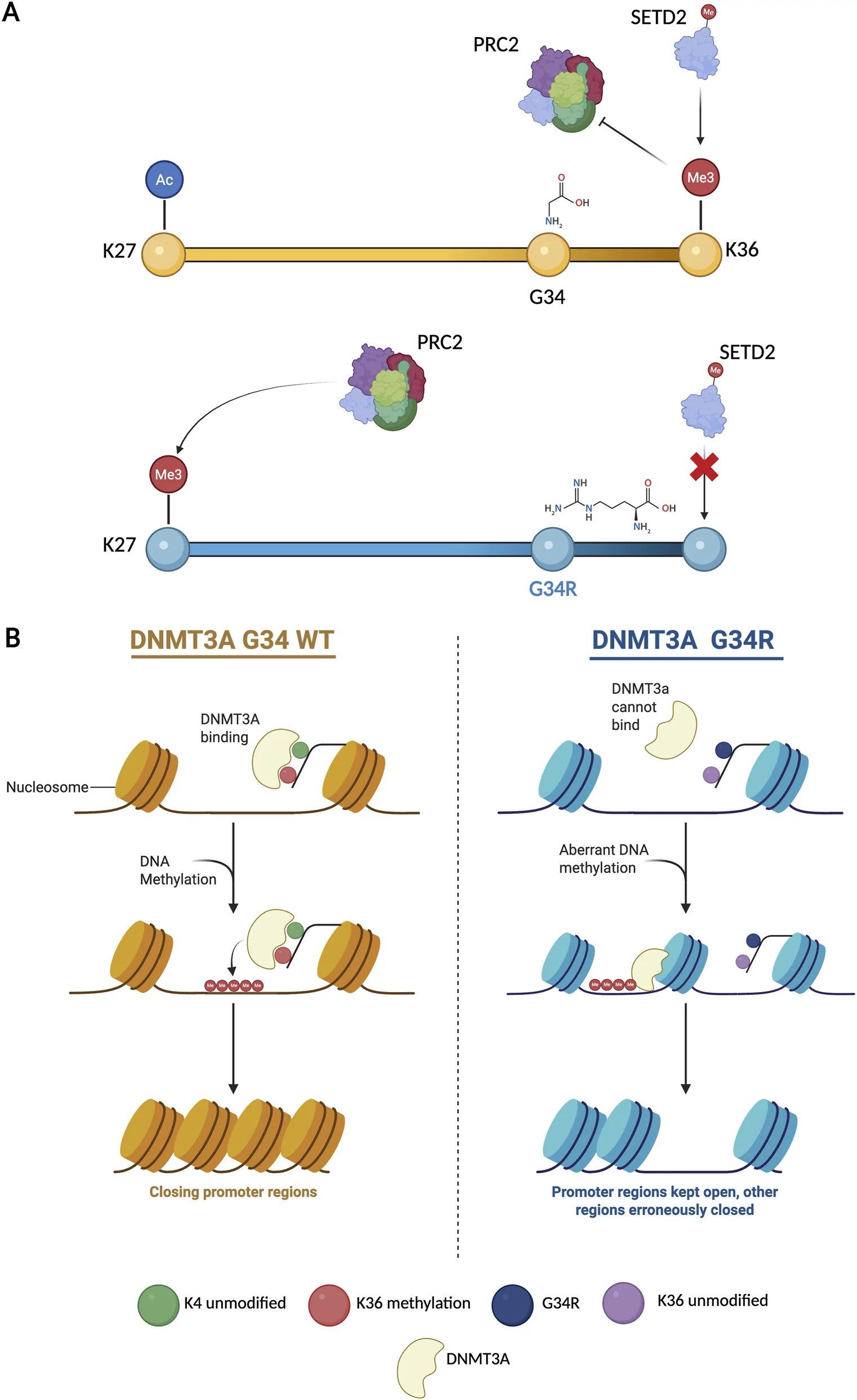
Epigenetic dysregulation resulting from H3.3 G34R mutation in DHG. **(A)** SETD2 mechanism of action in promoting methylation of the K36 residue is perturbed by allosteric inhibition of the G34R mutation. **(B)** DNMT3A binding is blocked indirectly by the G34R mutation through inhibiting K36 methylation and thereby not methylating DNA target regions and being displaced elsewhere. Created in BioRender. Barry et al. (2026) https://BioRender.com/tsborxi.

With all that is understood so far about the biology of this disease, this has not yet been translated to meaningful changes in survival rates. Some of the biggest questions regard the temporal paradigm of the H3.3 G34 mutation and the onset of clinical disease. These tumours present with a stalled differentiation phenotype with cells remaining in an embryonic interneuron precursor state, yet the patients present as AYAs where the main neurodevelopmental process that is happening is oligodendrogenesis and myelination. Questions that we still do not understand include do H3.3 G34R mutations occur early or later during cortex development? Is there a way to analyse the temporal dynamics of H3.3 G34R mutations and how do they potentially evolve over time? In the next section, we will discuss hPSC-derived 3D brain organoids and how the field is transitioning to this system for understanding disease aetiology and improving translational outcomes. We will discuss why these are suitable models for studying human epigenetic mechanisms and why their manipulability and accessibility may answer some of the aforementioned questions, making them very appropriate for studying DHG and pHGG more broadly.

## Human pluripotent stem cell (hPSC) derived 3D brain organoids to model compartments of the developing brain

hPSC-derived 3D brain organoids have emerged as a means to investigate human neurodevelopment due to the fidelity of genomic expression when compared to human foetal samples (Lancaster and Knoblich, 2014). The formation of 3D brain organoids commences from human pluripotent stem cells, which have the capacity to self-renew and differentiate into cells of the three germ layers (Takahashi and Yamanaka, 2006; Niwa et al., 1998; Thomson et al., 1998; Takahashi et al., 2007). hPSCs can form embryoid bodies, which are then guided to differentiate into the neuroectoderm with dual SMAD inhibition and manipulation of Wnt, SHH and FGF/EGF pathways (Birey et al., 2017; Paşca et al., 2022; Lancaster et al., 2013). hPSC-derived 3D brain organoids represent a desired subsection of the developing human brain grown in a dish formed by the directed differentiation of hPSCs. There are several different types of neural organoids that have been developed, representing different parts of the brain (Table 2). These can be used to understand biological mechanisms of different disorders as individual organoids or in specific neural circuits by fusing different types of organoids together, as assembloids (Figure 4). Most commonly, brain organoids have been used to model neuropsychiatric diseases like Timothy Syndrome (Birey et al., 2022), Parkinson’s Disease (Reumann et al., 2023), Autism Spectrum Disorder (ASD) (Rabeling and Goolam, 2023) and Schizophrenia (Dixon and Muotri, 2023). In particular, diseases with monogenic germline mutations can be modelled by using patient-derived induced PSCs (iPSCs) and differentiation protocols for specific brain regions. Germline mutations can be gene-edited to generate an isogenic control of the same patient genetic background. Techniques such as immunofluorescence, single cell sequencing and electrophysiology of brain organoids can then be used to infer links between phenotypes and the underlying biological mechanism.

**TABLE 2:** Table of current models of brain regions.

Organoid type	Brain region modelled	References	Disease modelled
Cortical organoid (hCO)	Embryonic dorsal forebrain (pallial)	(Paşca et al., 2015; Birey et al., 2017)	Timothy syndrome
Subpallial spheroid (hSS)	Embryonic ventral forebrain (subpallial)	(Birey et al., 2017)	Timothy syndrome
Cerebral organoid	Multiple developing brain regions	(Lancaster et al., 2013)	Microcephaly
Midbrain organoid	Ventral midbrain/dopaminergic neuron regions	(Jo et al., 2016)	-
	Midbrain-striatum-cortex assembloid	(Reumann et al., 2023)	Parkinsons disease
Arcuate organoids (ARCOs)	Hypothalamic arcuate nucleus	(Huang et al., 2020)	Prader-Willi Syndrome
Hippocampal organoid	Hippocampus	(Yu et al., 2014)	-
		(Pomeshchik et al., 2020)	Alzheimer Disease
Cerebellar organoid	Cerebellum	(Muguruma et al., 2015)	-
		(Ballabio et al., 2020)	Medulloblastoma
Optic-cup vesicles	Retina precursor cells/retinal pigment epithelium	(Meyer et al., 2011)	Gyrate atrophy (retinal degeneration)
Thalamic organoid (vTHO)	Ventral Thalamus	(Xiang et al., 2019)	-
		(Kiral et al., 2023)	Disease associated PTCHD1 or ERBB4 genes
Striatum organoids	Cortico-striatal pathway	(Miura et al., 2020)	Deletion on chromosome 22q13.3
Hindbrain/Cervical Spinal Cord spheroids	Cortico-motor assemblies	(Andersen et al., 2020)	-
Brainstem organoids	DMG and brainstem organoids with microglia to model the immune microenvironment	(Bessler et al., 2026)	Diffuse Midline Glioma (DMG)
		(Eura et al., 2020)	-
DMG-Cortical organoid co-culture	DMG patient derived cells co-cultured with cortical organoids as an assembloid and mosaic cultures	(Prior et al., 2024)	DMG

**FIGURE 4:**
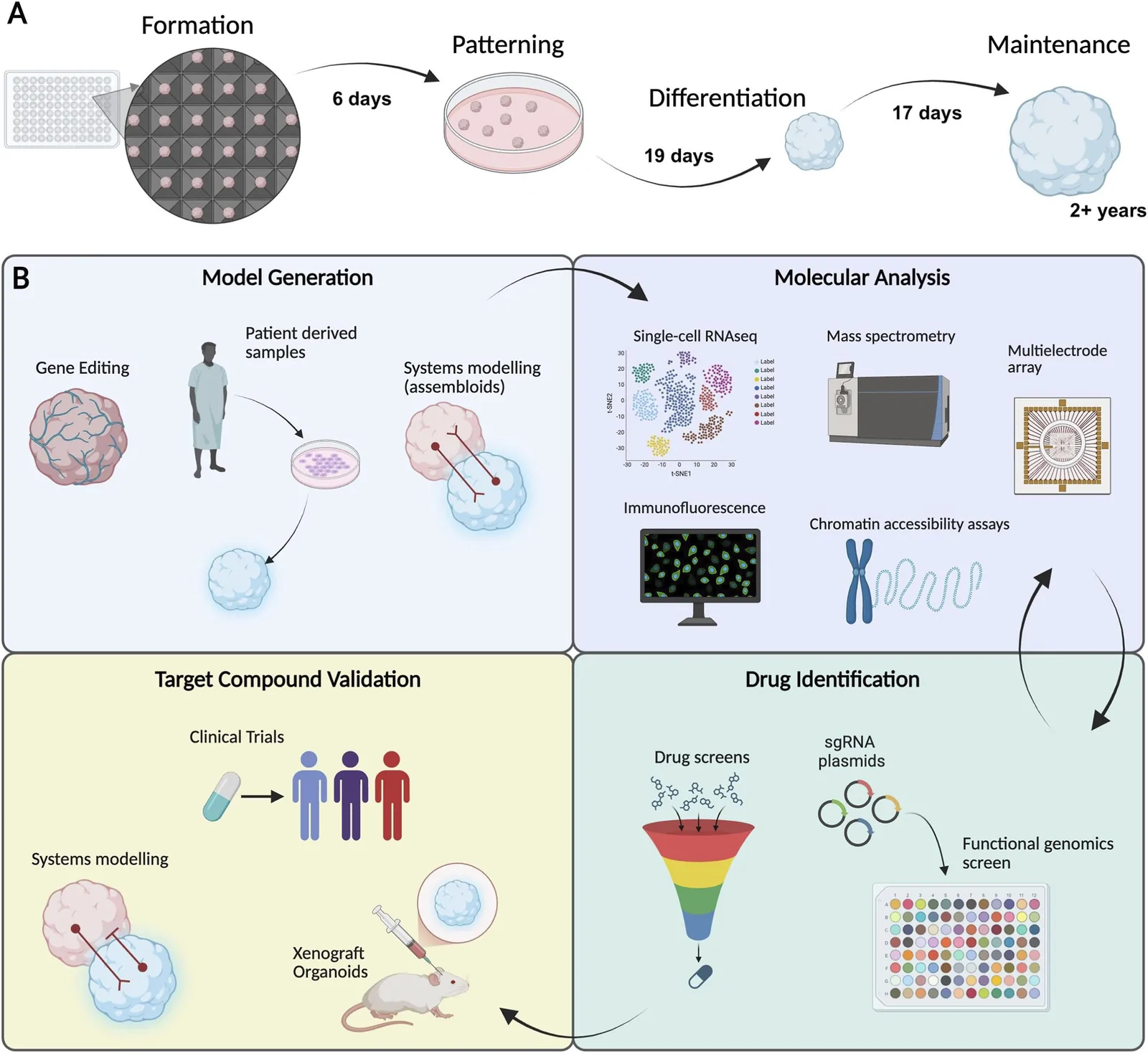
Human iPSC-derived brain organoids in discovery science. **(A)** Brain organoid growth follows 4 distinct phases: formation, patterning, differentiation and maintenance. Organoid formation usually begins with Dual SMAD inhibition to promote neural induction, signalling pathway agonists/inhibitors such as SAG (SHH) or IWP2 (WNT) to induce patterning and neurotrophic and growth factors such as BDNF and N2 to promote differentiation. Days between phases are based on the ventral/dorsal forebrain differentiation protocol from the Pasca Laboratory, as detailed in Birey et al. (2017). Length of phases differ between brain organoid models. **(B)** The cycle of discovery harnessing brain organoids for accurate disease modelling, molecular analyses, drug identification and validation of targets. Created in BioRender. Barry et al. (2026) https://BioRender.com/6k12seo.

In addition to modelling specific diseases, brain-region specific organoids have proved indispensable for understanding cell fate trajectories and neuronal migration during brain development. An example that is particularly relevant to the formation of the neocortex and the balance of inhibitory and excitatory neurons during forebrain development is the generation of ventral and dorsal forebrain organoids pioneered in the laboratories of Sergiu Pasca, Jurgen Knoblich and In-Hyun Park (Bajaj et al., 2021; Birey et al., 2017; Xiang et al., 2017). The tangential migration of newborn neurons from the subpallium to pallium is recapitulated *in vitro* by fusion of ventral and dorsal forebrain organoids to form an assembloid, which provides a more complex representation of the human developing forebrain. In this model, interneuron migration from the GE (ventral forebrain) to the developing neocortex (dorsal forebrain) can be visualised (Sloan et al., 2018).

## Examining disease mechanism and understanding with 3D brain organoids

Interneuron migration is important for disease mechanisms in conditions that rely on migration of GABAergic interneurons, production of glutamatergic neurons and formation of the cerebral cortex such as Timothy Syndrome, autism spectrum disorder and epilepsy. (Bajaj et al., 2021; Birey et al., 2017; Xiang et al., 2017). The electrical activity of ventral/dorsal assembloids have been shown to be biologically relevant as they contain both GABAergic inhibitory and glutamatergic excitatory neurons, and a readout of this activity can be measured by patch clamping slices of organoids or using a 3D multielectrode array (Birey et al., 2017; Sloan et al., 2018). Treatment of 3D Brain organoids with candidate drugs in culture provides a tractable means to measure the effects on parameters such as organoid size, cell growth, cell death, and expression of key markers and gene signatures (Bajaj et al., 2021). The 3D architecture of organoids accurately recapitulates human brain architecture and can serve as a proxy for drug screening and more specified trials (Park et al., 2021). Although the potential for drug screening is becoming more feasible with reproducible organoid protocols (Velasco et al., 2019), there is still the issue of organoid heterogeneity, batch effects in organoid generation, and quality control measures for organoids to meet the criteria needed for drug screening on a consistent basis.

3D organoid models are also valuable because of their capacity for high level molecular analysis in human tissue. High-resolution single cell analysis has enabled insight into the molecular profile of 3D brain organoids in comparison to human foetal samples to both validate their fidelity to *in vivo* human neurodevelopment and to provide detailed information on the transcriptome or chromatin organisation (Nowakowski and Salama, 2022). The ability to make inferences as to the spatial region and developmental timepoint and their alignment with human foetal development allows improved biological investigation of neurodevelopment (Herring et al., 2022). In general, the developmental timelines for hPSC organoids are not well defined (Heide et al., 2018). However, forebrain organoids at Day 70 show a similar transcriptional profile to the developing human forebrain at gestational week 18–20 (Birey et al., 2017). Similarly, it is reported that cerebral organoids recapitulate cell-type specific patterns of enhancer accessibility; however, comparison to *in vivo* counterparts reveals that they do not replicate accessibility to broader chromatin accessibility states (Ziffra et al., 2021). Although there may be discrepancies, the fact that human brain organoids can be benchmarked against human tissue make them valuable for understanding disease aetiology and biological mechanism with greater fidelity to human biology.

DHG, as described previously, is an epigenetic disorder. 3D brain organoids provide the appropriate neurodevelopmental context, in this instance utilising either ventral or dorsal forebrain organs as the source of migrating GABAergic interneurons, but they can also assist in the elucidation of epigenetic mechanisms. This is primarily centred around their accessibility. By accessibility we mean firstly that we can manipulate the genome through gene editing to recreate different epigenetic mutations (Bian et al., 2018). Secondly, we can readily perform experiments to assess changes in epigenetic states. For example, ATAC-seq, ChIP-seq, DNA methylation analysis (including bisulfite sequencing) and CUT&Tag or CUT&RUN sequencing assays all can be used to assess different functional components of epigenetic regulation (Uzquiano et al., 2022). As a result, functional expression differences can be subsequently analysed with scRNAseq for transcription or mass spectrometry for proteomic analysis to inform the expression outcomes from changes in epigenetic regulation (Koetsier et al., 2026). Particularly useful for DHG would be techniques like single nucleus multiome sequencing (snMultiome-seq), where we can correlate accessibility changes with snATAC-seq and changes in transcription (snRNA-seq) to determine cell type populations at the single cell level.

This overview of how hPSC-derived 3D brain organoid models recapitulate neurodevelopment and their accessibility for molecular analysis highlights why this model system may be very useful for investigating DHG. Specifically, because it can accurately model the human neurodevelopmental environment that the tumour cells recapitulate, that these cells can be manipulatable by drugs and gene editing (Bian et al., 2018), and molecular analyses can be performed to further explore the complex and relatively unknown epigenetic, transcriptional and cell signalling mechanisms at play in disease aetiology.

## Future of 3D brain organoids in pre-clinical validation of drugs and clinical translation for treatment of DHG

### How 3D brain organoids can be employed in clinical translation for DHG

Organoids are 3D tissue structures cultured *in vitro* derived from either adult stem cells, cancer tissue directly or hPSCs (e.g., embryonic stem cells or induced pluripotent stem cells) (Schutgens and Clevers, 2020). The latter is the subject of this review. hPSC-derived brain organoids provide a human model system that is accessible for screening new drug targets and drug compounds as well as modelling specific disease questions. In the context of a tumour sub-type such as pHGG tumour DHG, a rare tumour type with limited access to tissue samples, hPSC-derived brain organoid models offer a tractable model for investigation of disease aetiology, exploration of mechanism and drug discovery.

Brain organoids derived from PSCs have proved to be important in modelling disease. There are a number of examples of the use of organoids in modelling neurodevelopmental and neurodegenerative disorders, and cancer (Table 2). Autism Spectrum Disorder (ASD) has been investigated through cortical organoids. Research from Daniel Geschwind and Sergiu Pasca groups collected a cohort of 70 hPSC lines to compare divergent and convergent ASD traits. They identified a shared RNA and protein interaction network that converges on ASD risk genes identified in ASD cortical organoids to then validate by a functional CRISPR screen providing therapeutic opportunities (Gordon et al., 2026). Similarly, by employing calcium imaging and electrode recordings of 3D brain organoids derived from individuals with Rett Syndrome, Samarasinghe et al. (2021) demonstrate epileptiform activity. Using this model a physiological rescue experiment was performed with the neuroregulatory drug, pifithrin-α (Samarasinghe et al., 2021).

There are also multiple ways to model cancer using organoids. Several studies have co-cultured patient-derived tumour cells with iPSC-derived cerebral organoids to model tumour-brain interactions (Azzarelli et al., 2021; Lago et al., 2023; Linkous et al., 2019). This system allows scientists to track tumour metastasis and invasion in real time visually by microscopy and assess the effect of the tumour cells on neural behaviour and *vice versa*. Co-culture organoid models can also be used to test chemotherapies and targeted drugs and assess their effects on both the tumour cells and neural cells. A specific example detailed in (Prior et al., 2024) highlights how diffuse midline glioma (DMG), another sub-type of pHGG is modelled with co-culture of patient-derived tumour cells with cortical organoids derived from healthy iPSC donors. In this study they culture tumour spheres with cortical organoids as assembloids, two distant organoids or spheres fused together in culture, as well as mosaic cultures, which is dissociating both the tumour spheres and cortical organoids, mixing them, and culturing them as one.

Brain cancer organoids can also be used as patient avatars in drug screens to test targeted therapies and to investigate synergistic combinations with standard of care therapy such as radiation therapy and chemotherapies. Anne Rios’ group has designed hPSC-derived organoids to recapitulate the driver and co-mutations of DMG (Bessler et al., 2026). Here a piggyback system was used to express the H3K27M driver mutation along with PDGFRA and TP53 (Bessler et al., 2026). Through this model the authors were able to recapitulate the DMG phenotype in brainstem organoids and explore CAR-T therapies using co-culture of the DMG organoids with myeloid cells to create an immune microenvironment. These myeloid cells generated DMG-specific microglia that made the immunotherapy treatments less effective due to immunosuppression but more physiologically relevant. This highlights one of the limitations of organoids models; that they are lacking other non-neuronal cell types such as microglia and endothelial cells. Further, brain organoids lack a vascular supply, have a limited maturation time, and are not connected to functional neuronal systems. Some of these limitations are being overcome by co-culturing with other cell types, like the microglia cell co-culture system described above to replicate aspects of the immune microenvironment and vascularisation of organoids to imitate the blood brain barrier (Zhou et al., 2025; Bessler et al., 2026). Assembloids provide systems to model more complex systems such as the sensory and motor pathways (Kim et al., 2025; Andersen et al., 2020). In Table 3, we have detailed strengths and limitations of different model systems to complement the text and convey that every system has its strengths to best utilise in different applications.

**TABLE 3:** Strengths and Limitations of pHGG model systems.

Model system	Description	Strengths	Limitations
Genetically engineered mouse models (GEMMs)	Genetically altered mice expressing oncogenes or loss of tumour suppressor genes to create an oncogenic phenotype (Kersten et al., 2017)	Advantageous for drug toxicity testing and *in vivo* pharmacodynamics due to presence of functioning blood-brain barrier and immune microenvironment Clinically relevant tumour location (Furst et al., 2024)	Shorter lifespan of mice leads to mismatched correlation of treatment timing in GEMMs to human drug therapy and dosage (Ma et al., 2015; Zhavoronkov et al., 2025) Does not accurately capture human disease biology and structural architecture due to species differences Mouse cohorts are genetically identical (Zuberi and Lutz, 2016)
Patient-derived xenograft mouse models (PDX)	Transplantation of human derived tumour cells into immunodeficient mice ectopically or orthotopically (Xu et al., 2019)	Allows for assessment of drug delivery and therapeutic response in an environment with an intact blood-brain barrier Presence of microglia and neuroimmune environment despite immunodeficiency (Lorke et al., 2008)	Reduced efficacy of preclinical drug toxicity testing due to xenografts lacking structural networks supporting the tumour (Olive et al., 2009) Species differences in the immune landscape between humans and mice (Mestas and Hughes, 2004) Tumour transplantation can be spatially irrelevant to human tumour disease biology
Patient-derived cell lines	A primary cell culture line established from patient tumour biopsy or sample (Cheng et al., 2022)	Recapitulates patient tumour molecular heterogeneity and human disease background (Furst et al., 2024; Mirabelli et al., 2019) 3D model for drug screening and to assess therapeutic drug efficacy (Wong et al., 2017) Bypasses animal ethics barrier of conducting *in vivo* experiments (Mirabelli et al., 2019)	Lack of immune and tumour microenvironment (Mirabelli et al., 2019) Lack of networks with other cell types and tumour architecture (Mirabelli et al., 2019)
Patient-derived cancer organoids	Cultures of 3D tumour cell aggregates from patient-derived samples (Gunti et al., 2021)	Allows for genetic editing to model diseases akin to patient and human biology Allows for co-culture models with hPSC-derived tumours to explore tumour-brain interplay 3D model for drug screening and to assess therapeutic drug efficacy (Bessler et al., 2026) Recapitulates human brain architecture, vital for modelling human disease biology Bypasses ethical barrier with conducting in vivo experiments	Limited representation of the tumour microenvironment (Mirabelli et al., 2019) Variability due to disease and patient heterogeneity (Moore et al., 2025) Inadequate for modelling tumour origin and initiation
hPSC-derived organoids	Cultures of 3D cell aggregates derived from human induced pluripotent stem cells or embryonic stem cells (Vandana et al., 2023)	3D model for drug screening and to assess therapeutic drug efficacy (Bessler et al., 2026) Recapitulates human brain architecture, vital for modelling human disease biology Bypasses ethical barrier with conducting *in vivo* experiments Provides neurodevelopmental context which is important for pHGG biology. For example, dorsal or ventral forebrain organoids and/or assembloids of dorsal and ventral forebrain can be utilised Accessibility for molecular analyses such as scRNA-seq and ATAC-seq Electrophysiologically active, can detect differences using Microelectrode Arrays (Passaro and Stice, 2020)	Lacks non-neuronal populations such as glial cells, endothelial cells and immune cells (Jin et al., 2026) Lacks vasculature Organoid heterogeneity and batch effects with generation (Jensen and Little, 2023)

Although there is currently a gap in literature on using brain organoids specifically for DHG, the above examples with DMG provide precedent for what can be understood about pHGG dynamics through organoid models. As the H3.3 G34 driver mutation in DHG is a somatic mutation, patient derived iPSCs may not be as applicable comparatively to other neurological germline disorders. However, this can be addressed by co-culturing with patient tumour cells or complementing the H3.3 mutation with co-mutations to create a *de novo* cancer model. This is used to study disease-specific signatures, including antigen presentation and CAR-T; cancer metastasis; neural-tumour interactions; and RNA, protein and chromatin accessibility changes.

The ability to grow 3D brain organoids at scale and in high quantities has opened new possibilities for both functional genomics and drug screening opportunities. This has been aided by the design of machine learning algorithms and automated imaging to be able to track differences in parameters such as organoid growth, proliferation, morphology or a specific fluorescence readout (Ballabio et al., 2020; Lago et al., 2023; Ramani et al., 2024). Large-scale compound libraries with thousands of candidate drug compounds can been screened effectively to identify hits for further testing. Similarly, functional genomics screens have emerged to analyse susceptibilities or risk genes in tumours and neurodevelopmental disorders. For example, Meng et al. (2023) employed a functional genomics screen with 425 neurodevelopmental disorder risk genes in forebrain assembloids to identify 13 candidate genes involved in interneuron migration through tracking fluorescent nucleokinesis across assembloid boundaries (Meng et al., 2023).

3D brain organoids also provide a platform to trial new experimental targeted approaches for promising lead therapeutics. For example, an antisense oligonucleotide has been identified as a novel treatment of Timothy Syndrome as a result of experimental validation in the forebrain assembloid model (Birey et al., 2022; Chen et al., 2024). Similarly, cortical organoids have been used to uncover the mechanism of action in Rett Syndrome (Trujillo et al., 2021). Using patient lines with disrupted Methyl-CpG-binding protein 2 (MeCP2), scientists identified a compound called HRP-12975, which increases MECP2 protein expression and improves neuronal connectivity (Trujillo et al., 2021). The potential for discovery in 3D brain organoids from detailed mechanistic understanding to actionable therapeutics is revolutionary for scientific discovery and clinical translation. Effective policy and regulatory frameworks should accompany this technology to maximise the potential benefits in discovery, translation and economic activity.

### Transitioning from animals to new approach methodologies (NAMs)

Governments are actively shifting away from traditional animal models for preclinical testing. For example, the UK government has released a policy paper, describing their plans for “phasing out the use of animals in all but exceptional circumstances” (Department for Science, Innovation & Technology, Home Office, and Department for Environment, Food & Rural Affairs, 2025). Similarly, in 2025 the US Food and Drug Administration (FDA) released their ‘Roadmap to Reducing Animal Testing in Preclinical Safety Studies’ highlighting the exponential growth of New Approach Methodologies (NAMs), citing that at least 90% drugs that are approved safe in animals are not translated to approval for humans (U. S. Food and Drug Administration, 2025; Marshall et al., 2023). In Europe, a European Citizens Initiative submitted a report titled ‘Save cruelty-free cosmetics–Commit to a Europe without animal testing’ to the European commission which adopted recommendations to be released in the first quarter of 2026 as Europe’s roadmap to animal-free testing (European Commission, 2023).

These government responses reflect three factors: First, the public opinion on animal cruelty and recognising animals as sentient beings (European Commission, 2023). Second, the inadequate translatability of drugs in animal models to humans; a particular example is in cancer research, where the successful translation rate from animals to humans is as low as 8% (Mak et al., 2014). Lastly, the advent of NAMs that use human-specific biology in the form of *in vitro* assays with stem cells, organoids and organ-on-a-chip technology, along with *in silico* analyses has given a robust alternative to clinical translation. These technologies are accessible, less expensive, scalable and often faster than traditional animal preclinical validation (Driehuis et al., 2020; Liu et al., 2020; Ramani et al., 2024). Organoids are an important and necessary part of the future of discovery research for DHG and pHGG more broadly. The capacity to illuminate tumour formation mechanisms, screen candidate drugs and develop novel epigenetic and targeted therapies provides a route for pre-clinical validation and addressing the challenges of treating DHG for the benefit of adolescents and young adult patient outcomes.

### Concluding remarks

Here, we have described key neurodevelopmental trajectories in the development of the human cortex. Starting from the neural tube to the developing forebrain, we described how morphogen gradients along the SHH and Wnt pathways, along with key transcription factors such as DLX2 and GSX2, promote GABAergic interneuron cell fate. These processes are tightly orchestrated and maintained by epigenetic regulation. Epigenetic regulation by DNA methylation and histone PTMs, specifically on the H3.3 variant, is important in timing these developmental waves. These aspects of neurodevelopmental biology are fundamentally linked in DHG, an epigenetically dysregulated cancer affecting adolescents and young adults, and molecularly defined by a stalled interneuron differentiation phenotype. The emergence of hPSC-derived 3D brain organoids has become a tractable way to investigate human neurodevelopment in a dish. We discussed the fidelity of hPSC-derived 3D organoids to human tissue and the capacity for high level molecular analysis and drug screening capacity that can be translated to actionable therapeutic strategies. Organoid models have played an important role in allowing a shift in government and public opinion away from the use of animal models that can be used for improved therapeutic discovery in hard-to-treat diseases.

Growth in science is accompanied by creative destruction. We learn and experiment with models and ideas until they become outdated. Then we break them down and pivot to include new technologies and new models. This is an important process, for the more we understand human biology the more questions arise. This necessitates greater power and accuracy from our tools to answer them. For a disease like DHG, where the 5-year survival rate is ∼10–15% with minimal improvement over the past 3 decades, new model systems such as hPSC-derived 3D brain organoids may provide solutions to what has so far proven to be a complex problem. Use of human stem cell derived brain organoids may result in more human specificity, greater screening capacity, and faster translatability to achieve a mechanistic understand of disease aetiology towards identification of novel and more effective therapies.
